# Quantification of practice effects in a self-administered online cognitive pre-screening test

**DOI:** 10.1007/s10072-026-09333-5

**Published:** 2026-08-18

**Authors:** Hans-Aloys Wischmann, Willem Huijbers, Murray Gillies, Ziad Nasreddine

**Affiliations:** 1 Institute of Public Health, Charité - Universitätsmedizin Berlin, Charitéplatz 1, 10117 Berlin, Germany; 2 MoCA Cognition, Montréal, QC Canada

**Keywords:** Cognitive Screening, Mild Cognitive Impairment, Dementia, Practice Effects, Discrimination Power, Online

## Abstract

**Background:**

With growing prevalence of mild cognitive impairment and dementia, online self-administered pre-screening tests hold the promise to more objectively identify individuals who stand to benefit from prioritized access to supervised cognitive testing and in-depth neurological evaluation, while reducing the burden of unnecessary tests for physicians and the health care system.

**Objective:**

We aimed to quantify practice effects in same-day and mid-term test-retest applications of the XpressO cognitive pre-screening test, to develop novel methods to stratify practice effects in the absence of ground truth, to quantify the discrimination power in retests, and to incorporate information from practice effects to improve the discrimination power for retests.

**Methodology:**

We performed a retrospective observational study to analyse data from a large, self-enrolled online cohort. We created descriptive statistics for test-retest comparisons of all relevant performance metrics, developed an unsupervised machine learning approach to impute the cognitive status of participants, and used this to compare the discrimination power in the initial test-retests on the same day or after a gap of two weeks or more.

**Results:**

Significant practice effects were found in a self-enrolled online population, in same-day retests and after several weeks, with differentially larger effects observed for presumably cognitively impaired participants. Practice effects reduced the discrimination power of the original test score in the retests.

**Conclusion:**

Whenever participants can perform unlimited, uncontrolled, and unsupervised practice sessions, increased task variation is desirable for future cognitive pre-screening tests. Quantified practice effects contain independent, additional predictive information regarding the cognitive status of online participants.

**Supplementary Information:**

The online version contains supplementary material available at https://doi.org/10.1007/s10072-026-09333-5.

## Introduction

### Background

The prevalence of mild cognitive impairment (MCI) and dementia increases with age [1, 2], thereby representing a particular challenge in ageing populations. Timely disease detection and organization of appropriate patient care represent growing burdens for healthcare systems overall, but especially for primary care physicians, geriatrists, neurologists, and neuropsychologists. Delayed diagnoses and misdiagnoses exacerbate this issues [3]. At the same time, many older adults are very attentive to their health and develop concerns regarding their cognition once they notice new or increasing memory issues. In turn, this positive awareness increases the number of cognitively normal adults who desire a medical evaluation.

Cognitive pre-screening tests, especially self-enrolled and self-administered online tests, hold the promise to objectively identify individuals who stand to benefit most from seeking care from their family physician or general practitioner [4, 5]. Presumed patients could benefit from prioritized access to cognitive assessments, supervised cognitive testing, and in-depth neurological evaluation, while the overall burden of unnecessary diagnostic tests could be reduced.

However, in contrast to clinically-administered cognitive tests, such short pre-screening tools face several novel and critical challenges, including the unsupervised setting, the ability to repeat tests at will, the lack of ground truth regarding the true cognitive status of participants, and self-selection biases for the initial enrolment and retests. Especially the necessary brevity of a pre-screening test combined with participants’ ability to repeat it multiple times and thus practice all of its tasks may lead to a positive drift of test scores over time, which may negatively impact the test’s performance. Complementary information derived from quantified practice effects, stratified by demographic variables and cognitive status, may enable the creation of a “practice-aware score” that eliminates or mitigates this effect, both for repeated online self-test scenarios and for repeated pre-screening of the same patient in a physician’s practice, e.g., in preparation for annual wellness visits.

The unprecedented size of a self-enrolled online cohort promises detailed and robust insights regarding test scores at baseline as a function of sex, age, education level, and presumed cognitive status; quantified practice effects in test-retest applications over time; and the prevalence of presumed cognitive impairment among this online cohort, by sex, age, and education level.

### Research questions

The self-administered nature of online tests, combined with the possibility for participants to repeat the test at will, has raised several questions: (1) Are there practice effects in self-administered online cognitive pre-screening tests, in particular for the MoCA XpressO test [6]? (2) Which of the underlying test performance metrics are affected in short-term test-retest applications, and which practice effects persist after a longer gap between sessions? (3) Do the practice effects impact the discrimination power of the test score? (4) Do practice effects carry additional, independent information that can improve the discrimination power?

In contrast to learning effects that quantify improved (cognitive) skills that allow a participant to score better across a wider range of tests, practice effects relate to an improved performance when retaking the same (static) test immediately or after a gap, e.g., leading to shorter response times or to a higher number of correct answers for the same questions [7, 8].

### Research objectives

To address these questions, we aimed (1) to quantify practice effects in same-day and mid-term test-retest applications of the XpressO cognitive pre-screening test, for each of the relevant performance metrics, (2) to develop novel methods to stratify practice effects, in the absence of ground truth in an anonymous online cohort, using an imputed baseline cognitive status, (3) to quantify the discrimination power in retests, using this imputed baseline cognitive status as an outcome proxy, and (4) to incorporate information from the practice effects to improve the discrimination power.

## Methods

### Population

The study population has been described in detail elsewhere [9]: It consisted of persons who self-enrolled online between January 13, 2024, and March 31, 2025, and who subsequently took the XpressO test [6], either in a web browser or in an application that they installed onto a personal device. All participants consented electronically to the use of their anonymized data for research and development purposes. Neither (clinical) diagnoses regarding the cognitive status nor scores from (supervised) cognitive screening tests were available for the participants.

### Test-retest comparisons

The online instructions in the XpressO tool suggested to participants to return for a retest after a month, but it was possible to repeat the test immediately. Therefore, we analyzed both short-term practice effects, for participants who completed another test session on the day of their initial session, comparing the first completed follow-up session to their initial session, as well as mid-term practice effects, for participants who completed another session after a gap between sessions of 14 to 42 days, i.e., two to six weeks, comparing the first completed follow-up session after the gap to their initial session.

### Inverse probability weighting (IPW)

To compensate for potential self-selection bias in participants’ decisions to repeat the test, i.e., to complete an immediate or mid-term retest, we applied inverse probability weighting (IPW) in our test-retest analyses: We computed the probability of retaking the test on the same day or after a gap of 14 to 42 days, respectively, in a logistic regression model, as a function of the demographic variables sex, age, and education level, the platform (web browser or installed app) and the language (French or English), as well as the initial test performance, categorized as a Low (< 42), Intermediate, or High (> 72) XpressO score.

### Imputed cognitive status at baseline

While practice effects were expected in test-retest applications of the same cognitive pre-screening test, it was not known whether there would be differential effects for cognitively normal versus cognitively impaired participants, e.g., stronger in one group than in the other. As the self-enrolled online cohort was presumed to be dominated by cognitively normal participants, an unstratified analysis would have risked overlooking and underestimating the magnitude of any practice effects, especially in the minority group. On the other hand, an analysis stratified by the XpressO score in the initial session would have risked conceptual circularity, in analyzing changes in individual performance metrics stratified by an aggregate of the same metrics.

We therefore devised and applied a novel two-step unsupervised machine learning approach, using only performance metrics from the initial session of each participant, to independently characterize participants into two sub-populations that we tentatively labelled as cognitively normal versus cognitively impaired. For the initial step, we identified four concerns: (1) the observed overall test score and the individual test metrics are not only a function of cognitive status, but can also be influenced by sex, age, and education level, (2) some metrics as, e.g., the number of items placed in a particular test have a causal effect on others, including the number of correctly placed items and the test time, (3) degraded performance in memory tasks or in logical tasks may reflect independent impairments in different cognitive domains, and (4) continuous degrees of impairment may model participants’ status better than a single, dichotomous label. Structural Equation Modelling (SEM) allowed us to address all four of these concerns in a unified modelling framework. For the subsequent characterization step that partitioned participants into classes, we leveraged a Gaussian Mixture Model (GMM) approach because it allowed not only individual metrics to depend on demographic variables, but more importantly also allowed the probability of belonging to a particular class, and thus the probability of impairment, to depend on sex, age, and education level.

### Structural equation modelling (SEM)

First, cognitive status for the overall study population was therefore estimated on a continuous scale in a Structural Equation Model (SEM), based on observed item counts and question (response) time metrics from the XpressO test, separately for the memory and logical tasks. Baseline status was estimated as two distinct but potentially correlated, unobserved, latent continuous variables, to separately model status in cognitive domains relevant for memory and logical task performance, respectively, using data from the initial session only. Regressions were added for all metrics as a function of sex (dichotomous, converted to integer), age (continuous), and education level (ordinal, i.e., ordered categorical, converted to integer), and for the number of correct items and the question times, as a function of the number of items placed. The SEM specification is documented in the Supplementary Materials. Weighting (IPW) was not performed, as the initial session of all participants was analyzed.

### Gaussian mixture modelling (GMM)

Subsequently, cognitive status was estimated as a latent categorical variable in a Gaussian Mixture Model (GMM), based on the observed metrics from the XpressO test plus the two latent continuous cognitive status variables computed in the first step, using the SEM. Using the R package MoEclust [10, 11], influences of the covariates sex, age, and education level on the indicator variables and on the probability density were included. Models with 3 classes were computed, to separate a presumably cognitively normal sub-population from a probably cognitively impaired sub-population, while allowing for a third class, e.g., with intermediate or vastly superior cognitive performance.

Participants were tentatively classified as cognitively impaired (ML_CI) by this SEM-GMM unsupervised machine learning approach when they were categorized into the lowest memory performance class or into the lowest logical performance class, and otherwise as cognitively normal (ML_CN).

### Prevalence of cognitive impairment

We assessed the prevalence of presumed cognitive impairment in the overall study population based on this imputed classification, using a generalized additive logistic regression model (GAM) from the R package mgcv [12, 13], to create prevalence estimates stratified by sex and education level, with a smooth dependency on age, by sex and education level.

### Synopsis of XpressO scores

We subsequently leveraged the imputed cognitive classification to analyze the XpressO scores across all participants as a function of age, sex, education level, and tentative cognitive status, both to inspect the relationship between the imputed classification and the XpressO scores, and to create insights into the dependency of the XpressO scores on the demographic covariates. Here, we used another GAM to create a quantitative synopsis of the XpressO scores, for the initial session, again allowing for a smooth dependency on age, by sex, education level, and cognitive status. The parametric coefficients were used to quantify baseline XpressO score differences in the study population, i.e., offsets, for all education levels compared to primary education and for women compared to men.

### Quantification of practice effects

Practice effects were defined as the difference in observed metrics between the first completed retest session and the initial session, for each participant. Inverse probability weighting (IPW) was applied to adjust for self-selection bias in the participants’ decisions to retake the test, so as to estimate mean practice effects and standard deviations (SDs) that would have been observed if all participants had returned for both retests. Consequently, a weighted paired two-sample t-test was used to check each metric for significant differences, stratified by the imputed, tentative classification into cognitively normal versus cognitively impaired.

### Discrimination power

To assess the impact of practice effects on the discrimination power of the test, in the absence of clinically confirmed diagnoses or of scores from supervised cognitive screening tests, we used the dichotomous classification imputed by the unsupervised machine learning approach as a proxy for the cognitive status of each participant at baseline. Using data from the initial session, from all other tests on the day of the initial session, and from all tests completed after a gap of a two weeks or more, we quantified the discrimination power of the XpressO score that was previously presented in detail elsewhere [6, 9], using the receiver operating characteristic (ROC) and the area under the curve (AUC).

To explore whether the practice effects carried independent information that could support the classification task, we computed a generalized additive logistic regression model that predicted the imputed cognitive status at baseline as the outcome from the total question time, the total number of fully correct items in the logical test, and the total number of correct items in the memory test, as well as the respective difference in these three metrics compared to the initial session. In creating this practice-aware model, we used data from all sessions that were completed between 3 and 7 days after the initial session, to exclude both the initial session itself (from which the outcome is derived), as well as learning effects from the training sessions shortly thereafter. We evaluated the model’s discrimination power across all sessions completed more than 7 days after the initial session, i.e., on a different and non-overlapping subset of the data. The selection of the cutoffs (of 3 and 7 days, respectively) was arbitrary, and motivated only by a desire to exclude potential transitory effects in the initial, shortest-term retests and to include as many sessions as possible into complementary subsets for creation and validation of the model.

## Results

### Population

For the analyses in this study, we used the same dataset as in [9], which included only male and female participants, and excluded participants with implausible values in the self-reported years of post-kindergarten education, i.e., education years > age – 6 years. Additionally, we excluded all sessions for 351 participants older than 85 years, for 33 participants with incomplete data for their initial session, and for 36 participants with education levels that differed between sessions. No other sessions were excluded, all other data were complete. Years of education were categorized into Primary, Lower-Secondary, Upper-Secondary, Post-Secondary, and Tertiary education levels using cut-offs of 6, 10, 13, and 15 years, consistent with ISCED levels [14].

The study population is documented in Table 1, stratified by education level, showing the total population as well as the sub-populations of participants who completed another XpressO session on the day of their initial session or who completed another session after a gap of two to six weeks, i.e., between 14 and 42 days. Each stratum also shows the proportion of participants tentatively classified as cognitively impaired by the unsupervised machine learning classification approach, i.e., participants who were either in the lowest memory performance class or in the lowest logical performance class.

**Table 1:** Demographic characteristics of the study population and sub-populations with a retest on the same-day or after a gap of 2–6 weeks between sessions, overall and stratified by education level, with proportion of participants labelled as cognitively impaired by the machine learning approach (ML_CI)

Population	Education level	*N*	Mean age [min; max]	Female	XpressO 1st mean (SD)	ML_CI
Overall	Primary	1,371	61.4 [18; 85]	57.0%	55.4 (32.8)	10.9%
	Lower-Secondary	3,742	64.8 [18; 85]	56.7%	56.1 (32.6)	8.9%
	Upper-Secondary	13,121	62.9 [18; 85]	63.1%	65.1 (30.1)	7.3%
	Post-Secondary	9,385	59.9 [20; 85]	62.0%	71.0 (28.1)	5.8%
	Tertiary	24,141	56.8 [22; 85]	58.9%	75.9 (25.9)	4.7%
	Total	51,760	59.6 [18; 85]	60.3%	70.3 (28.8)	6.0%
Same-Day	Primary	250	59.5 [20; 84]	53.2%	66.9 (30.7)	16.8%
	Lower-Secondary	591	64.0 [18; 85]	53.1%	65.4 (31.2)	12.7%
	Upper-Secondary	1,927	61.8 [18; 85]	59.8%	72.1 (28.1)	11.7%
	Post-Secondary	1,436	59.5 [20; 85]	60.0%	76.1 (25.8)	10.2%
	Tertiary	4,066	57.6 [22; 85]	55.6%	79.1 (24.3)	9.1%
	Total	8,270	59.4 [18; 85]	57.1%	75.6 (26.6)	10.4%
2–6 Weeks	Primary	24	56.9 [22; 79]	75.0%	73.7 (24.9)	4.2%
	Lower-Secondary	54	62.0 [32; 82]	64.8%	77.4 (22.8)	5.6%
	Upper-Secondary	230	60.3 [18; 85]	69.1%	72.6 (27.2)	6.1%
	Post-Secondary	207	59.1 [20; 80]	68.6%	75.8 (26.8)	8.2%
	Tertiary	776	58.1 [22; 85]	61.2%	79.6 (25.2)	4.6%
	Total	1,291	58.8 [18; 85]	64.2%	77.5 (25.8)	5.5%

The analyzed study population comprised 51,760 participants, with a mean age of 59.6 years and a female majority (60.3%). Overwhelmingly, participants had completed upper secondary education or more. Overall, 6% were tentatively classified as cognitively impaired (ML_CI), with higher proportions among participants with lower education levels, reaching 9% to 11% for the strata of lower secondary and primary education, respectively.

The sub-population that repeated the test at least once on the day of their initial session was very similar to the overall study population in terms of mean age (59.4), with a slightly lower proportion of females (57.1%), but a higher proportion of ML_CI classifications, ranging from 9% for participants with tertiary education to 17% for participants with primary education.

The sub-population that repeated the test at least once after a gap of two weeks or more was again very similar to the overall study population in terms of mean age (58.8), with a slightly higher proportion of females (64.2%), and a lower proportion of ML_CI classifications, ranging from 4% to 8% across the education strata (with very few participants with primary or secondary education returning for a test after a longer gap).

### Inverse probability weighting (IPW)

Overall, the probability to return for at least one additional test on the day of the initial test was 16.0%, but only 2.5% for at least one additional test after a gap of 14 to 42 days. Women were significantly less likely than men to retake the test on the same day, participants with higher age were significantly less likely to retake the test on the same day and significantly more likely to retake the test after a gap of at least two weeks, and participants using the web browser or the French language were significantly less likely to retake the test. Regarding the language, this may be related to an enrolment wave after an article in a major newspaper in French [9]. Participants with higher XpressO scores in the initial session were less likely to retake the test on the same day, compared to participants with a low initial score: Some of the lower performing participants may have been dissatisfied with their initial achievements, and thus motivated to immediately repeat the test. This would be consistent with a higher fraction of probably impaired participants found in the same-day retest sub-population, compared to the study population, both overall and within each education bracket. Detailed odds ratios (ORs) are reported in the Supplementary Materials (see Table e1). Adequacy of the IPW model fits was verified by checking Variance Inflation Factors, visually inspecting decile-decile plots, and computing the discrimination power for predicting the return for a retest on the same-day (AUC = 0.703) or after a break (AUC = 0.818), where the AUC values indicated that additional factors influence participants’ decisions, beyond the covariates included in the logistic regression models.

### Prevalence of cognitive impairment

**Fig. 1:**
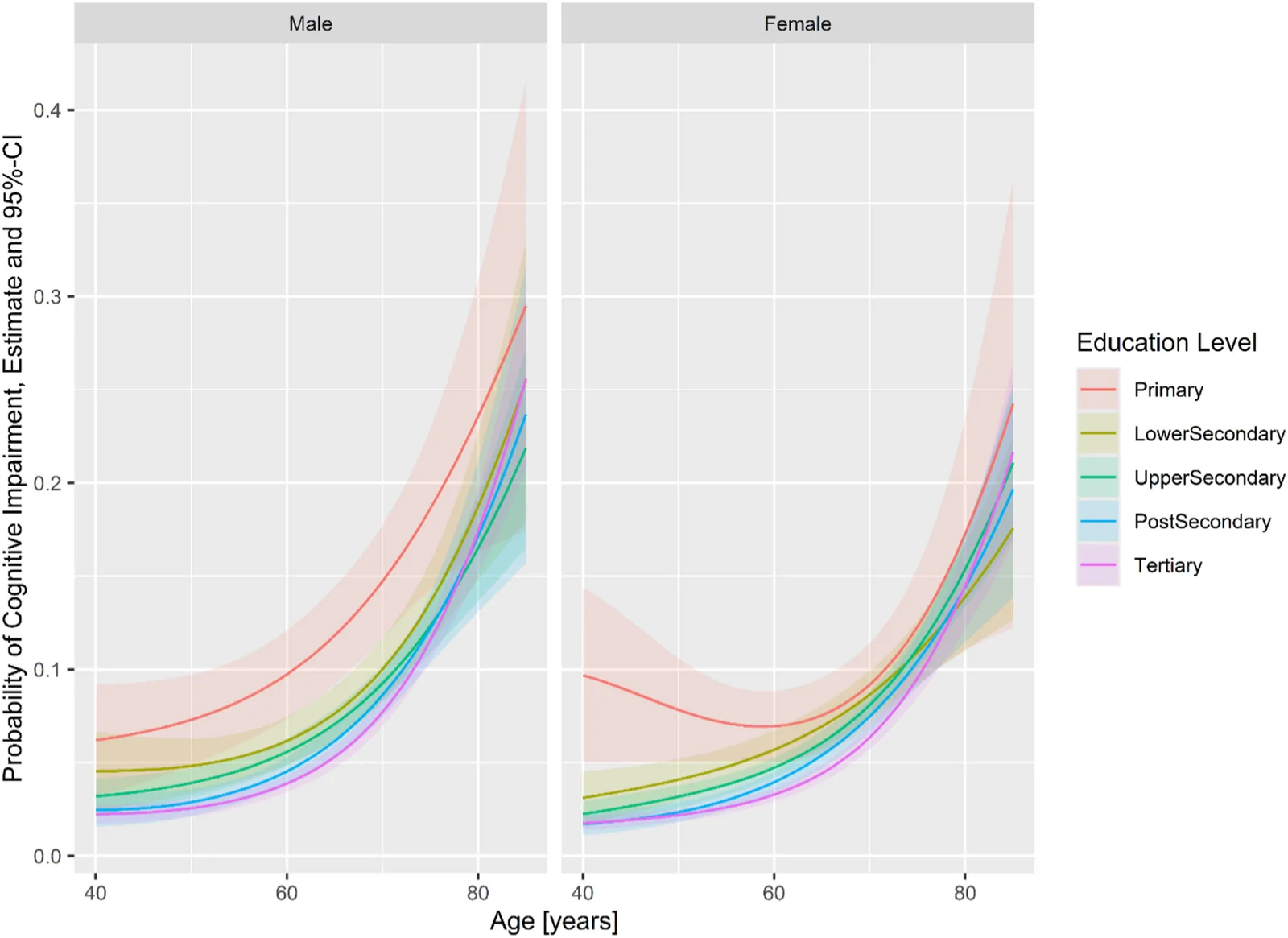
Estimated prevalence of cognitive impairment in the overall study population by age, stratified by sex and education level, from the generalized additive logistic regression model (GAM), with 95% confidence bands depicted as ribbons

Details regarding the prevalence of cognitive impairment in the overall study population, across sex and education strata are shown in Fig. 1, where a strong increase with age was found, starting from about 5% or less below the age of 50 years and reaching about 25% for men at age 85, with a somewhat lower prevalence among women than men of the same age, and a higher prevalence for participants with primary education, compared to participants with more education. Overall, across all strata and ages, an overwhelming majority of the study population was classified as cognitively normal by the unsupervised machine learning approach.

### Synopsis of XpressO scores

**Fig. 2:**
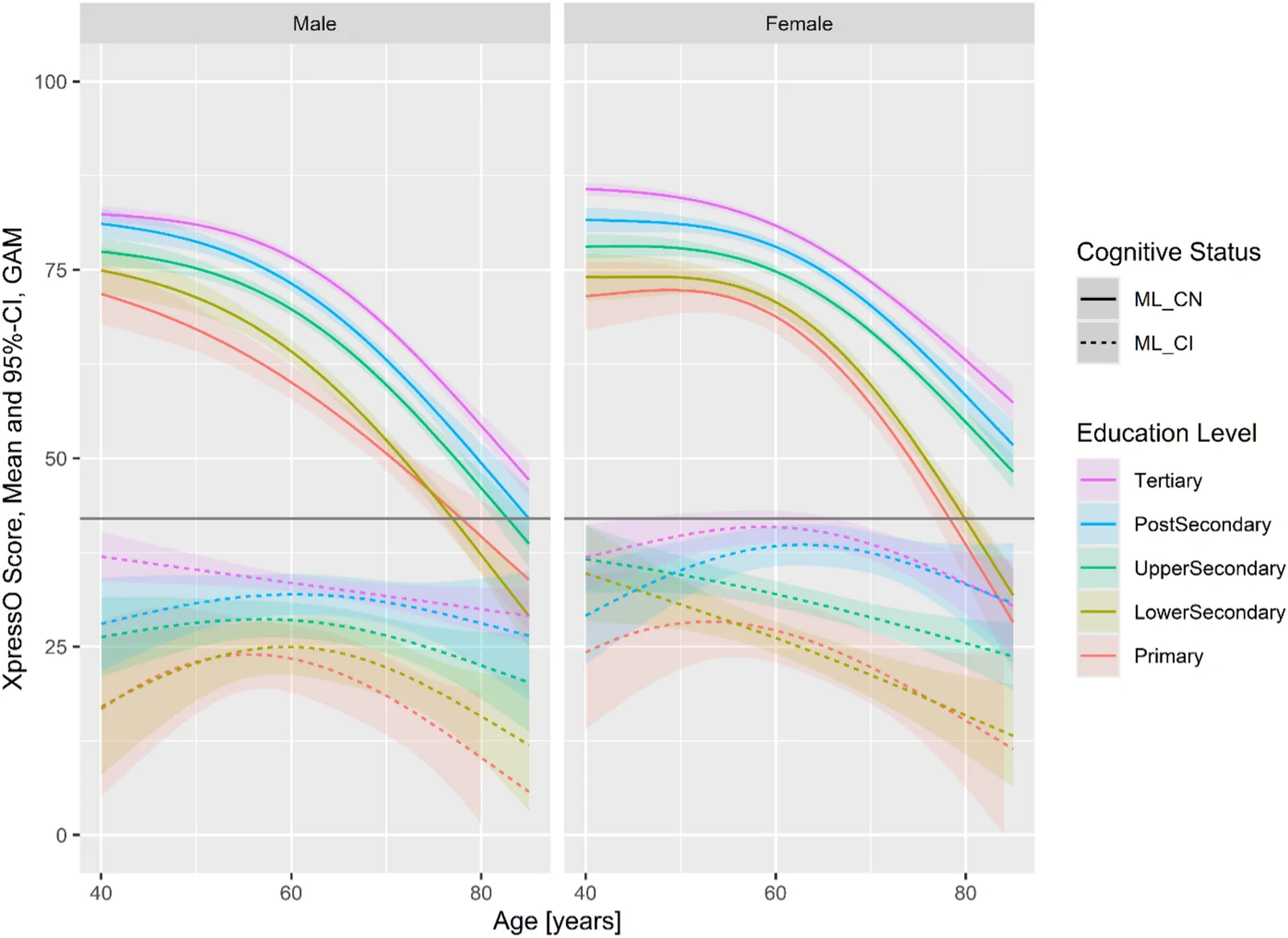
XpressO scores in the study population, for the initial session, stratified by sex, education level, and imputed cognitive status from the machine learning approach, with ML_CN indicating presumably cognitively normal participants, and ML_CI indicating probably cognitively impaired participants, from a generalized additive logistic regression model (GAM), with 95% confidence bands depicted as ribbons. The solid grey line shows the cut-off of 42 for a Low XpressO score

We quantified the relationship between the imputed cognitive status and the XpressO scores in the initial session: Details regarding the score, stratified by sex, education level, and cognitive status, are shown in Fig. 2, which exhibits a strong decrease of the XpressO score with age for cognitively normal participants, starting around the age of 50, slightly earlier for men than for women, and lower scores for lower levels of education. For cognitively impaired participants, the observed XpressO scores were much lower, typically below the threshold of 42, again with lower scores for lower levels of education and for ages above 50. However, the uncertainty margins were larger due to the smaller number of participants that were classified as cognitively impaired. The regression coefficients for the parametric variables demonstrated that differences for sex, education levels, and imputed cognitive status were all statistically significant, as reported in the Supplementary Materials (see Table e2).

### Quantification of practice effects

For participants who were categorized by the unsupervised machine learning approach as cognitively impaired (ML_CI), all performance metrics showed statistically significant practice effects for same-day retests, and all metrics related to the logical tests showed statistically significant effects also for mid-term retests. In particular, these participants performed retests much faster than in their initial session, with an (IPW-weighted) average speed-up by 2 min or more; they placed 1 to 2 additional items in the memory test, 2 additional items in the logical test, and increased the number of correct items, colors, and positions even more, by up to 4 additional correct items in the logical test, as documented in Table 2.

For cognitively normal participants (ML_CN), the performance metrics in the initial session were higher and response times were shorter than for cognitively impaired participants (ML_CI), and all response times and all performance metrics for the same-day retests showed statistically significant practice effects. However, the differences compared to their initial session were much smaller than for cognitively impaired participants, including speed-ups by up to only 1 min. The number of items placed decreased by statistically significant but small amounts, and the change in the number of correct items and positions was also small (or even slightly negative).

Comparing the first retest after a gap of at least two weeks to the first same-day retest, cognitively normal participants thus appeared to plateau or fall back slightly, consistent with a wash-out of the practice effects over time. By contrast, cognitively impaired participants continued to improve both their speed and their performance in the logical tests, consistent with a higher number of practice sessions performed prior to the first mid-term gap, but they appeared to plateau and fall back in their memory performance.

**Table 2:** Practice effects observed in XpressO retests on the same day of the initial session, or after a gap of 2–6 weeks between sessions, showing weighted mean differences in metrics, using weights proportional to the inverse probability for the respective retest (IPW). Statistically significant differences, with a p-value of less than 0.05 in an IPW-weighted two-sample paired t-test, are shown in bold, not statistically significant differences are shown in italic

Metric	Impaired (ML_CI)		Normal (ML_CN)	
	Same Day	2-6 Weeks	Same Day	2-6 Weeks
total question time [min]	-2.4	-2.0	-0.9	-0.6
logical question time [min]	-1.4	-1.6	-0.4	-0.3
memory question time [min]	-0.3	*-0.2*	-0.2	-0.1
logical number of items placed	2.0	2.3	-0.1	-0.1
logical number of 100correct items	2.8	4.3	1.2	*0.3*
logical number of correct colors	2.4	2.7	0.6	*0.1*
memory number of items placed	1.7	0.8	-0.1	-0.1
memory number of correct items	2.0	*0.8*	-0.1	-0.2
memory number of correct items and positions	2.4	*1.2*	0.1	*-0.1*
number of prior sessions	1.0	3.9	1.0	2.9

Detailed practice effects for each performance metric over the course of the first six XpressO sessions are depicted in the Supplementary Materials Figure e1, which shows the unweighted means as well as the average number of days between consecutive sessions, stratified by the imputed cognitive status.

To address potential concerns related to the low number of participants with primary education who returned for a retest after a longer break, a sensitivity analysis for the practice effects was performed, excluding participants with primary education. The results were mostly unchanged and never differed by more than 0.1 min or 0.1 item, as documented in the Supplementary Materials Table e3.

### Discrimination power

**Fig. 3:**
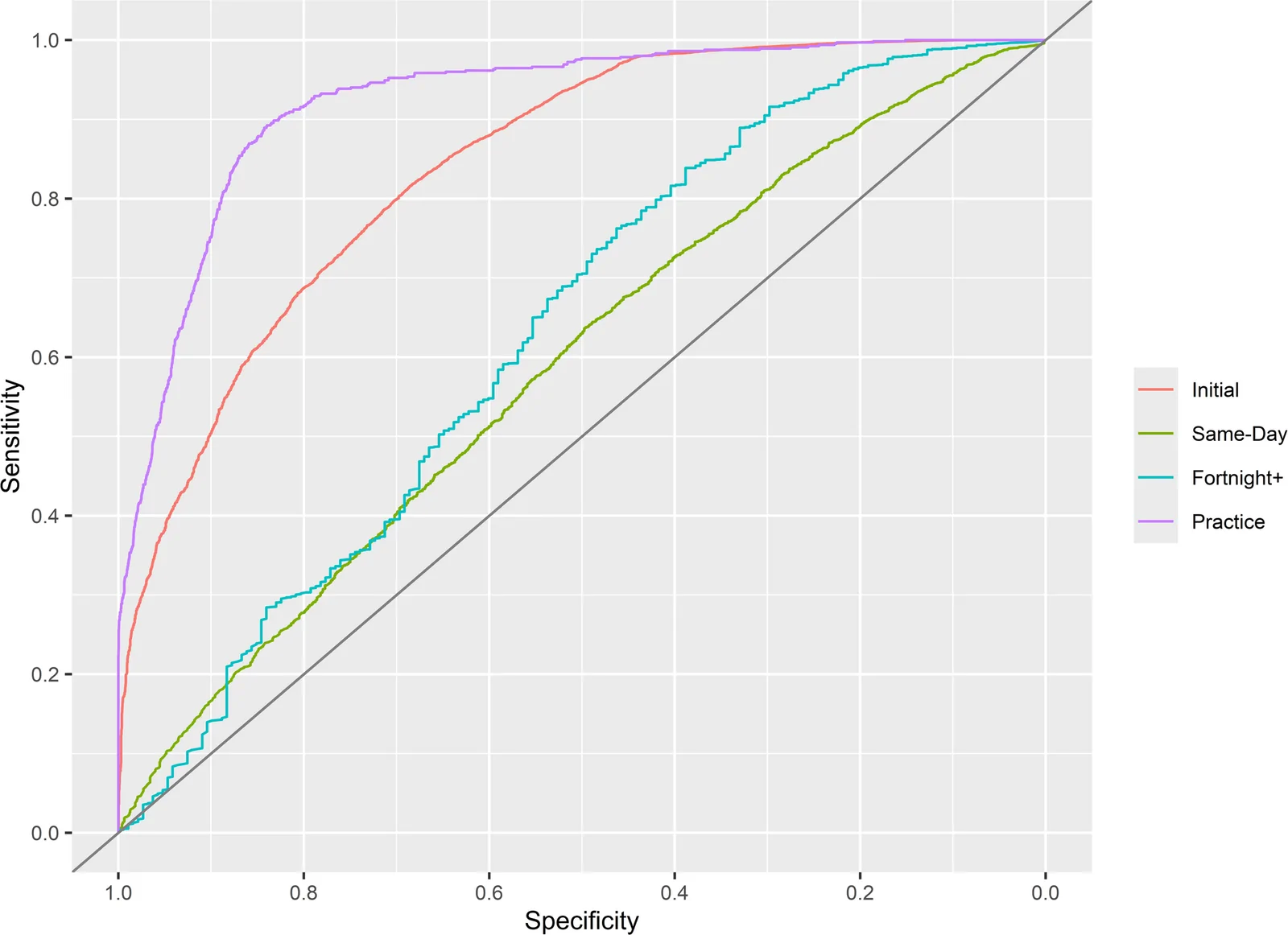
Receiver operating characteristic (ROC) curves for the XpressO score, evaluated for the initial session of each participant in red, for all retests completed on the same day in green, and for all retests completed after a gap of at least two weeks in blue. The ROC for the practice-adapted model, evaluated for all retests completed at least seven days after the initial session, is shown in purple

For the initial session, the XpressO score performed well in predicting the dichotomous classification from the unsupervised machine learning approach, as shown in Fig. 3, with an AUC of 0.842, comparable to the value that was reported in the original study [6]. However, its discrimination power dropped significantly for same-day retests, to only 0.589, and recovered very little after a gap of at least two weeks between tests, to 0.631.

However, when practice effects, i.e., differences in three metrics between the current session and the initial session were included into an updated scoring model, the discrimination power was statistically significantly superior (using DeLong’s test) with an AUC of 0.923, for all sessions that were completed at least one week after the initial session. For a desired sensitivity of at least 84%, which is appropriate for a pre-screening test setting, a specificity of 87% was found.

## Discussion

The unsupervised machine learning approach tentatively classified participants into cognitively impaired versus normal, so that practice effects could be stratified in the absence of a clinical diagnosis. The estimated prevalence of cognitive impairment in the self-enrolled online cohort was found to depend on age, sex, and education level, consistent with previous reports [9], but the machine-learning results aligned more closely with population estimates [15, 16].

With increased practice, all participants performed the XpressO test faster, but the speed increase was greater among cognitively impaired participants. The largest increases materialized between the first and second session, consistent with typical practice curves [17].

The number of items placed and the number of correct items increased more among cognitively impaired participants, especially in the logical task. However, this is partially due to the capped performance for cognitively normal participants, as the number of items to place and hence the number of correctly placed items is limited. Also, cognitively impaired participants may initially experience greater anxiety or difficulties with the online environment and instructions. Overcoming such challenges over the course of retest sessions would allow them to place more items and to place them correctly. This mirrors reported practice effects for the paper-based MoCA, where individuals with lower initial scores tended to improve more [18].

We found a wash-out of practice effects, consistent with studies that found smaller practice effects after a longer gap between test and retest [19]. For cognitively impaired participants, wash-out effects for logical tasks were presumably overshadowed by further improvements with additional practice sessions before taking their first longer break.

Since the individual metrics of test speed, number of items placed, and number of items placed correctly all improved for same day retests in both groups, i.e., also among participants with higher initial performance and in spite of ceiling effects, further improved for logical tasks for the probably impaired group and otherwise showed a plateau, it appeared unlikely that these effects could be due to a regression to the mean, so that we identified them as practice effects.

Practice effects were expected in repeated applications of the same online cognitive pre-screening test, and significant practice effects were found in the self-enrolled study population. Whenever participants can perform unlimited, uncontrolled, and unsupervised practice sessions, as in the setup of our study, increased task variation is therefore highly desirable for future cognitive pre-screening tests, to avoid misclassifications: As these practice effects reduced the difference in metrics between the cognitively normal and cognitively impaired participants, the discrimination power for the retest score was reduced. However, the discrimination power was improved significantly when practice effects relative to the first session were included into a modified practice-aware score, together with the observed performance metrics from the current session. Quantified practice effects thus contain complementary, predictive information regarding a participant’s cognitive status, which should be leveraged for future scoring algorithms.

### Strengths, limitations, and future work

The size and diversity of the online cohort enabled investigations that would be impossible in a traditional validation study. However, the associated lack of ground truth regarding participants’ cognitive status posed a challenge that was only partially overcome using a novel unsupervised machine learning approach. The imputed classification was useful as a stratification variable in quantifying differences in practice effects between presumably healthy and probably cognitively impaired subgroups, but the exploratory assessment of the discrimination power (AUC) suffered from a potential circularity, as the imputed status was derived from test metrics that also enter the calculation of the XpressO score. Future work should therefore compare the imputed cognitive status to self-reported diagnoses among online participants, and to clinical diagnoses in validation studies.

As another limitation, the self-enrolled online cohort was dominated by participants with upper secondary, post-secondary, or tertiary education, presumably overrepresenting healthy participants but potentially also participants with subjective cognitive decline. While the initial test was completed by more than 1,300 participants with primary education or less, (very) few of them completed a same-day retest or a retest after a longer gap. Although this unfortunately limits the generalizability of our results to this segment of the population, the share of persons with less than lower secondary education has been shrinking across many developed countries over the past decades. Future studies will thus require dedicated efforts to recruit a sufficient number of participants with only primary education or less, and of participants that are not native speakers of English or French, to support generalizability to other regions and countries.

### Conclusions

Significant practice effects were observed in repeated applications of the same online cognitive pre-screening test, both for same-day retests and for retests after a gap of several weeks, for all relevant performance metrics.

An unsupervised machine learning approach discriminated well between presumably cognitively normal participants and probably cognitively impaired participants, using only data from the participants’ initial test. Differentially larger practice effects were found for cognitively impaired participants, partially related to ceiling effects for unimpaired participants. This reduced the performance differences in the test metrics between unimpaired and impaired participants. As a consequence, practice effects reduced the discrimination power of the retest score, pointing to a need for varied tasks and stimuli in future cognitive pre-screening tests, whenever participants can perform unlimited, uncontrolled, and unsupervised practice sessions.

However, when practice effects relative to the first test session were included into a modified score, together with performance metrics from the current session, the discrimination power improved significantly, well beyond the power found using data from the initial test only. Practice effects were thus found to contain independent, additional predictive information regarding the cognitive status.

## Supplementary Information

Below is the link to the electronic supplementary material.

Supplementary Material 1 (DOCX 333 KB)

## Data Availability

The R code can be found in github at: https://github.com/wischmha/PracticeEffects.
