# Supplementary material for: Quantification of practice effects in a self-administered online cognitive pre-screening test
Source: Neurol Sci. 2026 Aug 18;47(9):713. doi: 10.1007/s10072-026-09333-5 (PMC13481588; doi:10.1007/s10072-026-09333-5)
Supplement: Supplementary file 1 [file 10072_2026_9333_MOESM1_ESM.docx]

**Supplementary Information**

**Journal**

Neurological Sciences

**Title**

Quantification of Practice Effects in a Self-Administered Online Cognitive Pre-Screening Test

**Authors**

Hans-Aloys Wischmann^1^, Willem Huijbers^2^, Murray Gillies^2^, Ziad Nasreddine^2^

^1^ Institute of Public Health, Charité – Universitätsmedizin Berlin, Berlin, Germany

^2^ MoCA Cognition, Montréal, Quebec, Canada

**Corresponding author**

Hans-Aloys Wischmann, Dr. rer. nat., ORCID: 0009-0000-1201-343X

Institute of Public Health, Charité - Universitätsmedizin Berlin, Charitéplatz 1, 10117 Berlin, Germany

hans-aloys.wischmann@charite.de

**Figures**

*Figure e1: Mean performance metrics stratified by session number and by cognitive status, as imputed by the machine learning (ML) approach, with ML_CN indicating presumably cognitively normal participants, and ML_CI indicating probably cognitively impaired participants.*

**Tables**

*Table e1: Odds ratios (ORs) with 95% confidence intervals (CIs) for completing another XpressO test on the same day as the initial session or after a gap of 2-6 weeks between sessions, as a function of sex, age, education level, and initial test score, from a logistic regression model.*

| Covariate | OR [95%-CI] Same day | OR [95%-CI] 2-6 Weeks | p-Value Same day | p-Value 2-6 Weeks |
| --- | --- | --- | --- | --- |
| Female vs. Male | 0.90 [0.86, 0.95] | 1.09 [0.97, 1.22] | <0.001 | 0.17 |
| Age [+10 years] | 0.98 [0.96, 1.00] | 1.22 [1.17, 1.28] | 0.014 | <0.001 |
| Lower-Secondary vs. Primary | 1.00 [0.84, 1.18] | 1.25 [0.77, 2.08] | 0.98 | 0.37 |
| Upper-Secondary vs. Primary | 0.98 [0.85, 1.15] | 1.33 [0.88, 2.10] | 0.84 | 0.19 |
| Post-Secondary vs. Primary | 1.09 [0.94, 1.28] | 1.48 [0.98, 2.33] | 0.27 | 0.079 |
| Tertiary vs. Primary | 1.09 [0.94, 1.26] | 1.43 [0.96, 2.22] | 0.28 | 0.096 |
| Intermediate vs. Low (XpressO) | 0.94 [0.88, 1.00] | 1.00 [0.83, 1.20] | 0.050 | 0.99 |
| High vs. Low (XpressO) | 0.26 [0.24, 0.28] | 0.90 [0.77, 1.06] | <0.001 | 0.21 |
| Web Browser vs. App | 0.49 [0.46, 0.52] | 0.10 [0.08, 0.12] | <0.001 | <0.001 |
| French vs. English | 0.67 [0.63, 0.72] | 0.55 [0.47, 0.64] | <0.001 | <0.001 |

*Table e2: XpressO score differences and 95% confidence intervals (CIs) for the initial session, as a function of education level, sex, and imputed cognitive status, from a generalised additive regression model (GAM) with smooth functions of age by sex and by education level.*

| Covariate | Score Difference [95%-CI] | p-Value |
| --- | --- | --- |
| Lower-Secondary vs. Primary Education | 2.5 [0.9, 4.1] | 0.002 |
| Upper-Secondary vs. Primary Education | 8.2 [6.7, 9.6] | <0.001 |
| Post-Secondary vs. Primary Education | 11.6 [10.2, 13.1] | <0.001 |
| Tertiary vs. Primary Education | 15.0 [13.6, 16.4] | <0.001 |
| Female vs. Male | 4.9 [4.5, 5.4] | <0.001 |
| ML_CN vs. ML_CI | 39.7 [38.6, 40.8] | <0.001 |

*Table e3: Practice effects observed in XpressO retests on the same day of the initial session, or after a gap of 2-6 weeks between sessions, showing weighted mean differences in metrics, using weights proportional to the inverse probability for the respective retest (IPW), as in Table 2, but excluding participants with primary education. Statistically significant differences, with a p-value of less than 0.05 in an IPW-weighted two-sample paired t-test, are shown in bold, not statistically significant differences are shown in italic.*

|  | Impaired (ML_CI) | | Normal (ML_CN) | |
| --- | --- | --- | --- | --- |
| Metric | Same Day | 2-6 Weeks | Same Day | 2-6 Weeks |
| total question time [min] | **-2.4** | **-2.0** | **-0.9** | **-0.6** |
| logical question time [min] | **-1.4** | **-1.6** | **-0.4** | **-0.3** |
| memory question time [min] | **-0.3** | *-0.2* | **-0.2** | **-0.1** |
| logical number of items placed | **2.0** | **2.3** | **-0.1** | **-0.1** |
| logical number of 100correct items | **2.8** | **4.3** | **1.2** | *0.3* |
| logical number of correct colors | **2.5** | **2.7** | **0.6** | *0.1* |
| memory number of items placed | **1.7** | **0.8** | **-0.1** | **-0.1** |
| memory number of correct items | **2.0** | *0.7* | **-0.1** | **-0.2** |
| memory number of correct items and positions | **2.5** | *1.1* | **0.1** | *-0.1* |
| number of prior sessions | 1.0 | 3.9 | 1.0 | 2.9 |

**Structural Equation Model (SEM)**

In SEMs, a latent variable can only be estimated up to a shift and an arbitrary, irrelevant scale factor (and thus sign). The direction and scale of the imputed status in either cognitive domain is therefore fixed by setting the regression factor to 1.0 for the number of fully correct items in each domain. The model is specified using the SEM syntax of the R package lavaan [1] as follows, where mem and log are latent, unobserved variables for the cognitive status regarding memory and logical tasks, respectively, sex, age, and edu_level are demographic variables, and variables starting with sum are performance metrics from the execution of the XpressO cognitive screening test:

mem =~ 1.0 * sum_memory_number_of_correct_items_and_positions +

sum_memory_number_of_correct_items +

sum_memory_number_of_items_placed +

sum_memory_question_time

log =~ 1.0 * sum_logical_number_of_100correct_items +

sum_logical_number_of_correct_colors +

sum_logical_number_of_items_placed +

sum_logical_question_time

sum_memory_number_of_correct_items_and_positions +

sum_memory_number_of_correct_items +

sum_memory_number_of_items_placed +

sum_memory_question_time +

sum_logical_number_of_100correct_items +

sum_logical_number_of_correct_colors +

sum_logical_number_of_items_placed +

sum_logical_question_time ~

sex + age + edu_level

sum_memory_number_of_correct_items_and_positions +

sum_memory_number_of_correct_items +

sum_memory_question_time ~

sum_memory_number_of_items_placed

sum_logical_number_of_100correct_items +

sum_logical_number_of_correct_colors +

sum_logical_question_time ~

sum_logical_number_of_items_placed

mem ~~ log

**Supplementary References**

1.	Rosseel Y (2012) lavaan: An R Package for Structural Equation Modeling. Journal of Statistical Software 48:1–36. https://doi.org/10.18637/jss.v048.i02
